# A New Anæsthetic

**Published:** 1869-04

**Authors:** 


### A New Anæsthetic,

Or, rather, an improvement upon an old one, is proposed by Dr. E. Andrews of Chicago. A recent number of the *Chicago Medical Examiner* contains a paper by Dr. Andrews, giving the results of some experiments with the nitrous oxide in association with certain quantities of free oxygen. The object of these experiments was to ascertain if, by mixing oxygen with nitrous oxide gas, it might not have conferred upon it a *sustaining power*, so as to be applicable to the longest surgical operations. It is well understood that, while nitrous oxide acts promptly, and leaves none but the most agreeable sensations behind, its healthy anæsthetic action cannot be very long continued. For use in extracting teeth, and for minor surgical operations, it is better and more desirable than either ether or chloroform; but for the more serious surgical cases, requiring the patient to be kept insensible for a long time, it has been found to produce unpleasant and dangerous symptoms.

The theory regarding the cause of these effects is, that the oxygen is so firmly locked up in the combination that it cannot aid in the revivification of the blood, and hence is as inert as the oxygen of carbonic acid. The nitrous oxide needs to hold in mechanical combination sufficient oxygen to sustain life while it is acting to produce anæsthesia. Dr. Andrew's claims that his experiments prove that *oxygen-mixed nitrous oxide* is an agent perfectly safe to use in the longest as well as the shortest surgical operations, and that it forms the pleasantest and best anæsthetic known. He recommends one volume of pure oxygen to be mixed with three of nitrous oxide, and remarks that care must be used, in administering it, that no atmospheric air becomes mingled with it.—*Boston Journal of Chemistry.*

